# Cdk2: a key regulator of the senescence control function of Myc

**DOI:** 10.18632/aging.100140

**Published:** 2010-04-25

**Authors:** Per Hydbring, Lars-Gunnar Larsson

**Affiliations:** ^1^ Department of Microbiology, Tumor and Cell Biology (MTC), Karolinska Institutet, 171 77 Stockholm, Sweden; ^2^ Present address: Department of Cancer Biology, Dana-Farber Cancer Institute, Boston, MA 02115, USA

**Keywords:** Myc, cyclin-dependent kinase 2 (Cdk2), Ras, oncogene-induced senescence, cell cycle

## Abstract

Proto-oncogenes such as MYC and RAS promote normal cell growth but fuel tumor development when deregulated. However, over-activated Myc and Ras also trigger intrinsic tumor suppressor mechanisms leading to apoptosis and senescence, respectively. When expressed together MYC and RAS are sufficient for oncogenic transformation of primary rodent cells, but the basis for their cooperativity has remained unresolved. While Ras is known to suppress Myc-induced apoptosis, we recently discovered that Myc is able to repress Ras-induced senescence. Myc and Ras thereby together enable evasion of two main barriers of tumorigenesis. The ability of Myc to suppress senescence was dependent on phosphorylation of Myc at Ser-62 by cyclin-dependent kinase 2 (Cdk2), uncovering a new non-redundant role of this kinase. Further, utilizing Cdk2 as a cofactor, Myc directly controlled key genes involved in senescence. We speculate that this new role of Myc/Cdk2 in senescence has relevance for other Myc functions, such as regulation of stemness, self-renewal, immortalization and differentiation, which may have an impact on tissue regeneration. Importantly, selective pharmacological inhibition of Cdk2 forced Myc/Ras expressing cells into cellular senescence, highlighting this kinase as a potential therapeutic target for treatment of tumors driven by Myc or Ras.

## Apoptosis and cellular senescence are two main barriers to cancer development

Cancer is in part driven by aberrant activation of growth-promoting oncogenes. However, overstimulation of cell growth by oncogenic signals causes severe cellular stress, which induces intrinsic tumor suppressor mechanisms resulting in apoptosis or cellular senescence [1]. Apoptosis is a programmed, energy-dependent cellular suicide process [2], while cellular senescence is a state of irreversible cell cycle arrest occurring in active, viable cells as a result of telomere erosion, DNA damage, hypoxia, oncogene activation or aging. Telomere erosion leads to replicative senescence as cells exhaust their proliferative capacity, while acute stress caused by oncogene activity can lead to premature, so called oncogene-induced senescence.

Cellular senescence has been demonstrated in different types of premalignant tumor cells *in vivo* during recent years, and has emerged as an important tumor-suppressive mechanism [3,4,5,6,7].

*MYC* and *RAS* are two prototypic oncogenes involved in development of numerous cancers. *MYC* encodes a pleiotropic transcription factor [8,9], while *RAS* codes for a signal-transducing GTPase [10]. However, Myc and Ras affect the failsafe mechanisms mentioned above in quite different ways. While Myc primarily induces apoptosis, Ras usually triggers cellular senescence. Myc-induced apoptosis is brought about though activation of p19Arf [11], which controls turnover of the tumor suppressor protein/transcription factor p53. p53 is a main executor of both apoptosis and senescence by controlling a large number of genes involved in these processes [12]. Myc can also induce apoptosis by suppressing anti-apoptotic members of the Bcl-2 family [9]. Mutations interfering with apoptotic signaling/execution are strongly selected for in Myc-driven tumorigenesis.

Ras on the other hand mainly triggers oncogene-induced senescence, which also involves upregulation of p53 via Arf [13,14]. In this case, anti-proliferative p53 target genes dominate, including p21Cip1, an inhibitor of cyclin-dependent kinase 2 (Cdk2) and Cdk1. Independent of p53, Ras also upregulates another Cdk inhibitor, p16Ink4, which targets Cdk4/6. The Cdks are the engines that drive the cell cycle by phosphorylating various substrates involved in this process. For instance, Cdk4/6 and Cdk2 target and inactivate the retinoblastoma tumor suppressor protein (pRb), an important "gatekeeper" that controls G1-S phase transition [15]. The Arf/p53/p21 and p16/pRb pathways therefore cooperatively regulate induction of senescence, and usually both of these pathways need to be intact for maintaining the senescent state [6,7].

In addition, Myc and Ras can also cause DNA damage, for instance by generating replication stress or reactive oxygen species (ROS) [13,16,17,18]. This induces DNA damage responses (DDR) that in turn can trigger apoptosis or cellular senescence. Depending on the type of lesion, DDR can cause activation of p53 via pathways involving the checkpoint kinases ATM and Chk2 or ATR and Chk1, which in turn induces p53 [19,20].

It is still unclear why oncogenic stress sometimes cause apoptosis and in other situations senescence. A possible explanation why Ras usually triggers senescence is that it activates the PI3/Akt kinase pathway, which dampens apoptotic signaling by inhibiting GSK3 kinase, FoxO and Bcl-2 family proteins [21]. Conversely, the reason why Myc primarily induces apoptosis may be because it directly or indirectly represses the p21 and p16 Cdk inhibitors as well as several anti-apoptotic genes [22,23,24,25,26], thereby possibly favoring apoptosis over senescence.

## Myc suppresses Ras-induced senescence with the help of Cdk2

**Figure 1.**
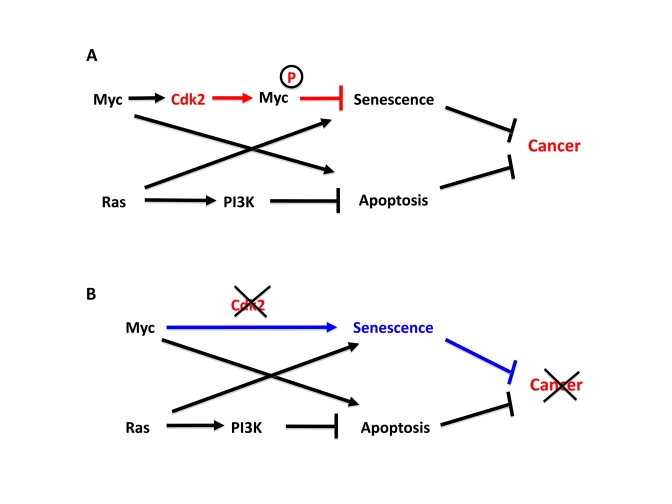
Cdk2 controls suppression of cellular senescence by Myc. (**A**) Apoptosis and senescence are two intrinsic tumor suppressor mechanism that are triggered by oncogenic signals. Myc and Ras contributes to this by inducing apoptosis and senescence, respectively. However, activated Myc and Ras are together sufficient to transform primary rodent cells for unclear reasons.  We recently found that while Ras suppresses Myc-induced apoptosis, Myc is able to suppress Ras-induced senescence, thereby together overcoming two main barriers of tumorigenesis. In order to suppress senescence Myc needs to be phosphorylated by Cdk2. Since Myc stimulates Cdk2 activity, Myc and Cdk2 are involved in an auto-stimulatory loop generating suppression of senescence. (**B**) Depletion or inhibition of Cdk2 abolishes to ability of Myc to suppress senescence and switches Myc into an activator of senescence, thereby inhibiting tumor development or maintenance.

It has been known since the early 80s that the combined activities of Myc and Ras are sufficient for oncogenic transformation of primary rodent cells [10]. The basis for the cooperativity has, however, remained unclear. One possibility is that they complement each other's capacity to induce mitogenic signals. Another possibility, that we have been exploring, is that the two oncogenes cooperate in suppressing the intrinsic tumor suppressor mechanisms described above. Ras is known from previous work to be able to suppress Myc-induced apoptosis through the PI3K/Akt pathway [27]. We recently demonstrated that Myc can repress Ras-induced senescence in primary rat embryo fibroblasts [24]. This suggests that at least part of Myc and Ras cooperativity is based on a "cross-pollination" mechanism where Myc abrogates the predominating tumor suppressing activity of Ras and vice versa, thereby together overriding two major barriers to tumor development (Figure 1A). Myc has previously been implicated in suppressing replicative senescence, which is caused by telomere erosion, by activating transcription of *hTERT* [28]. *hTERT* encodes a major component of telomerase, which is normally expressed in stem cells and prevents telomere shortening, thereby extending the replicative lifespan of cells.  Deletion of this gene inhibits Myc-driven lymphoma development, correlating with increased senescence [29]. Further, there are several reports demonstrating that reduced Myc levels induce cellular senescence in different settings. Lowering the Myc level by heterozygous knockout triggered telomere-independent senescence in human fibroblasts [26]. An elegant report using mouse tumor models with regulatable Myc showed that Myc shut-off caused regression of lymphomas, osteo-sarcomas and hepatocellular carcinomas, primarily as a result of increased cellular senescence [30]. Moreover, knockdown of Myc caused senescence in BRAF^V600E^- or NRAS^Q61R^-driven melanoma cells, while overexpression of Myc suppressed BRAF^V600E^ -induced senescence in primary melanocytes, [31]. Taken together, these reports suggest that Myc suppresses oncogene-induced as well as replicative senescence.

How does Myc suppress oncogene-induced senescence? Our recent work suggests that Cdk2 plays an important role in this regulation [24]. We could show that Cdk2-mediated phosphorylation of Myc at Ser-62 was crucial for bypassing Ras-induced senescence (Figure 1A). Interestingly, Cdk2 bound and phosphorylated Myc at promoter regions of target genes involved in regulation of cellular senescence such as *p21*, *p16*, *BMI1*, *CYCLIN D2* and *hTERT*. This correlated with low expression of *p21* and *p16* and high expression of *BMI1*, *CYCLIN D2* and *hTERT* [24]. The former and latter categories of genes are linked to activation and suppression of senescence, respectively. Importantly, inhibition of Cdk2 activity by selective pharmacological compounds or through interferon-γ-mediated upregulation of the endogenous Cdk inhibitor p27Kip1 abolished Ser-62 phosphorylation. This correlated with induced expression of *p21* and *p16*, repressed expression of *BMI1*, *hTERT* and *CYCLIN D2* and induction of senescence [24] (Figure1B). These results  suggest that Myc utilizes Cdk2 as a cofactor to directly control key genes in the p53/p21 and p16/Rb pathways and is thereby able to suppress senescence.

Although MAPK and Cdk1 are also reported to target Ser-62 [32], the anti-senescence function of Cdk2 could not be compensated by these kinases for unclear reasons. One explanation for this unique function of Cdk2 could be that it potentially also target additional proteins associated with Myc-regulated transcription, such as other transcription factors, cofactors or chromatin regulating proteins. Further, one cannot exclude that the Cdk2 function at chromatin synergizes with other unique but non-redundant, non-transcriptional functions of Cdk2, such as phosphorylation of p27.

What is the function of phospho-Ser-62 in suppression of senescence? Phosphorylation of Ser-62 is known to prime for GSK3-mediatied phosphorylation of Thr-58, which regulates the apoptosis function of Myc [33] and also its ubiquitylation and degradation [32]. However, a T58A mutant had no effect on senescence, suggesting that senescence regulation is a new and independent role of Ser-62. We did observe reduced association of Myc with target promoters upon Cdk2 inhibition, indicating that phospho-Ser-62 stabilizes Myc binding to chromatin. This is consistent with the work of Benassi et al [34], which demonstrated that MAPK-mediated phosphorylation of Ser-62 increased association of Myc to the γ-GCS gene in response to oxidative stress. Another plausible option is that phospho-Ser-62 provides an interaction surface for recruitment of a cofactor that participates in regulation of senescence-related Myc target genes.

In a parallel investigation together with the lab of Bruno Amati, we examined the impact of Myc alone, i.e. in the absence of other activated oncogenes, on cellular senescence in murine embryonic fibroblasts (MEFs). Strikingly, Myc activation resulted in senescence induction in Cdk2 knockout but not in wt MEFs, suggesting that Cdk2 suppresses Myc-induced senescence [35] (Figure 1B). Since Cdk2 function in the cell cycle is compensated by other Cdks during development [15], this is a unique, non-redundant role of Cdk2. It has been shown previously that Myc can induce senescence in cells lacking the Werner syndrome protein (WRN), a helicase implicated in DNA repair [36]. In Cdk2-/- cells, Myc-induced senescence was dependent on intact Arf-p53/p21 and p16^INK4a^-pRB pathways, and seemed to involve DDR [35], i.e. essentially the same pathways engaged by Ras [13,19,20]. One possible interpretation of the combined Hydbring and Campaner results is that Cdk2 and Myc constitute a senescence switch; Myc acts as a repressor of senescence when Cdk2 is active, but will provoke induction of senescence when Cdk2 is inactive (Figure 1). However, it is unclear at present whether the role of Cdk2 in suppression of Myc- and Ras-induced senescence is similar or distinct. Interestingly, both Cdk2 and WRN have been implicated in DNA repair [36,37], and could thereby possibly play a role in prevention and termination of the persistent DDR signaling that characterizes oncogene-induced senescence. Since Myc upregulates expression of WRN and hTERT and stimulates the activity of Cdk2 [8,28,36], it is conceivable that these proteins are part of an auto-protective loop that Myc uses to suppress senescence, perhaps at multiple levels. It will be important for future studies to identify Cdk2 substrates (other than Myc) that are essential for this process.

Regardless of mechanism, selective pharmacologic inhibitors of Cdk2, but not of other Cdks, forces embryonic fibroblasts with deregulated Myc or Myc/Ras into senescence [24,35] (Figure 1B). Further, Cdk2 ablation induced senescence in both pancreatic β-cells and hematopoietic B-cells after Myc activation, the latter correlating with delayed lymphoma development. This underscores that Cdk2 inhibitors should be reassessed as therapeutic agents, especially for Myc- or Ras-driven tumors. Cdk2 may be particularly suited for pharmacologic intervention since its function in the cell cycle is compensated by other Cdks in normal cells [38]. Previously, inhibition of Cdk1 was demonstrated to induce apoptosis in Myc-transformed cell but not in cells transformed with other oncogenes, and caused regression of Myc-driven tumors [39]. Therefore inhibition of Cdk1, Cdk2 or combination of these regimens should be considered in future treatment of Myc driven tumors based on molecular diagnosis of genetic and epigenetic status of intrinsic tumor suppressor systems of the tumor cells (Figure 1B).

## How does senescence regulation relate to other functions of Myc?

**Figure 2.**
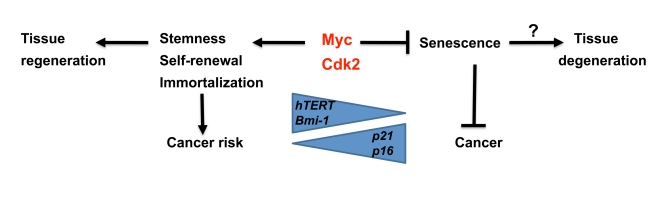
Speculative model illustrating regulation of senescence vs stemness and self-renewal by Myc and Cdk2. While Myc is a suppressor of senescence together with Cdk2, it stimulates stemness, self-renewal and immortalization, thereby potentially favor tissue regeneration. This is accomplished by activation of *hTERT* and *Bmi1*, and repression of *p21* and *p16*, key genes also implicated in regulating aging. The trade off for this capacity is increased risk for cancer development.

Cellular senescence is defined as an irreversible exit from the cell cycle - a feature that it shares with terminally differentiated cells. Myc's suppression of senescence therefore in many ways resembles its well-known function in inhibiting of terminal differentiation, which occurs in most (but not all) studied cell types [8,9]. One might consider inhibition of senescence and differentiation versus immortalization, self-renewal and tissue regeneration as two sides of the same coin (Figure 2). Myc is known since many years to contribute to immortalization of cells, for instance by supporting hTERT expression. Further, an increasing amount of data during recent years suggests that Myc is involved in the regulation of stemness. c-Myc and N-Myc, another Myc-family member, are essential for maintaining pluripotency and self-renewal of embryonic, hematopoietic and neuronal stem cells and progenitors [40,41,42]. This could also have an impact on processes like aging and aging-related diseases. Although the relation between cellular senescence and aging is far from clear, increased cellular senescence within stem cell populations could be one factor contributing to reduced tissue regeneration with age. In support of this, telomerase-deficient mice as well as mice expressing an overactive mutant of p53 have a significantly shortened lifespan [43,44,45]. DNA damage as well as p16Ink4a expression is known to increase with age, correlating with decreased tissue renewal and loss of stem cell function. This has been confirmed by deletion or overexpression of p16 in hematopoietic stem cells, forebrain neuronal progenitors and pancreatic β-cells [43,46,47,48]. The increased expression of p16 in aging tissues may in part be the result of decreased expression of Bmi1 [49], a major repressor of the p16Ink4a locus (Figure 2). One should note that Bmi1 is positively regulated by Myc [24,26].

This raises the question whether Myc and Cdk2 contribute to renewal and regeneration of adult tissues under normal conditions, and from this point of view could be considered as anti-aging factors? On the other hand, expression of these genes occurs at the expense of increased cancer risk and may therefore not result in increased longevity (Figure 2). It also raises the question whether combating Myc- and Myc/Ras-driven tumors with Cdk2-inhibitors comes with the side-effects of stem cell failure, decreased regeneration capacity in normal tissues and increased aging?  We think this is rather unlikely since Cdk2 inhibition in our hands induced senescence only in primary cells overexpressing Myc or Myc/Ras, but had little effect on untransfected cells.

One should also note that other reports do not support the notion that pro-senescence/anti-proliferative factors decrease regeneration capacity or promote aging under normal regulation. The proliferation rate of stem cells is normally kept low to protect them from the potential hazards of increased metabolism and cell division, and too high proliferation rate may lead to stem cell depletion, favoring the view that pro-senescence factors may have a protective role for stem cells [50].

In support of this notion, mice carrying an extra copy of the *p53* or *Arf/Ink4* loci display increased protection against cancer without any change in aging [43], and mice carrying an additional copy of both these loci displayed delayed aging, correlating with decreased aging-related DNA-damage [51], suggesting that Myc/Cdk2 inhibition not necessarily would have a negative impact on tissue regeneration and longevity. Interestingly, exogenous expression of hTERT in mice carrying the extra p53/Arf/p16 loci delayed aging even further [52].

In conclusion, there seems to be an intricate balance between anti- and pro-senescence factors in the regulation of stem cell capacity, regeneration of tissues and aging. The impact of Myc and Cdk2 inhibition both on tumor regression through senescence and potential effects on regenerative capacity of normal tissue needs to be addressed in mouse models in the future.
